# Early and Late Postoperative Seizures in Meningioma Patients and Prediction by a Recent Scoring System

**DOI:** 10.3390/cancers13030450

**Published:** 2021-01-25

**Authors:** Peter Baumgarten, Mana Sarlak, Daniel Monden, Andrea Spyrantis, Simon Bernatz, Florian Gessler, Daniel Dubinski, Elke Hattingen, Gerhard Marquardt, Adam Strzelczyk, Felix Rosenow, Patrick N. Harter, Volker Seifert, Thomas M. Freiman

**Affiliations:** 1 Department of Neurosurgery, University Hospital, Goethe University Frankfurt, 60528 Frankfurt am Main, Germany; mana-s@hotmail.de (M.S.); daniel.monden96@googlemail.com (D.M.); andrea.spyrantis@kgu.de (A.S.); florian.gessler@kgu.de (F.G.); daniel.dubinski@kgu.de (D.D.); gerhard.marquardt@kgu.de (G.M.); v.seifert@em.uni-frankfurt.de (V.S.); Thomas.Freiman@med.uni-rostock.de (T.M.F.); 2 Neurological Institute (Edinger Institute), University Hospital, Goethe University Frankfurt, 60528 Frankfurt am Main, Germany; simon.bernatz@kgu.de (S.B.); patrick.harter@kgu.de (P.N.H.); 3 Department of Neuroradiology, University Hospital, Goethe University Frankfurt, 60528 Frankfurt am Main, Germany; elke.hattingen@kgu.de; 4 Epilepsy Center Frankfurt Rhine-Main, University Hospital, Goethe University Frankfurt, 60528 Frankfurt am Main, Germany; strzelczyk@med.uni-frankfurt.de (A.S.); felix.rosenow@kgu.de (F.R.); 5 Department of Neurology, University Hospital, Goethe University Frankfurt, 60528 Frankfurt am Main, Germany; 6 LOEWE Center for Personalized Translational Epilepsy Research (CePTER), Goethe-University Frankfurt, 60528 Frankfurt am Main, Germany; 7 German Cancer Consortium (DKTK) Partner Site Frankfurt/Mainz, 60528 Frankfurt am Main, Germany; 8 German Cancer Research Center (DKFZ), 69120 Heidelberg, Germany; 9 Frankfurt Cancer Institute (FCI), 60528 Frankfurt am Main, Germany

**Keywords:** anticonvulsants, epilepsy, meningioma, seizures, STAMPE2

## Abstract

**Simple Summary:**

Seizures are among the most common symptoms of meningioma patients even after surgery. This study sought to identify risk factors for early and late seizures in meningioma patients and to evaluate a modified version of a score to predict postoperative seizures on an independent cohort. The data underline that there are distinct factors identifying patients with a high risk of postoperative seizures following meningioma surgery which has been already shown before. We could further show that the high proportion of 43% of postoperative seizures occur as late seizures which are more dangerous because they may happen out of hospital. The modified STAMPE2 score could predict postoperative seizures when reaching very high scores but was not generally transferable to our independent cohort.

**Abstract:**

Seizures are among the most common symptoms of meningioma. This retrospective study sought to identify risk factors for early and late seizures in meningioma patients and to evaluate a modified STAMPE2 score. In 556 patients who underwent meningioma surgery, we correlated different risk factors with the occurrence of postoperative seizures. A modified STAMPE2 score was applied. Risk factors for preoperative seizures were edema (*p* = 0.039) and temporal location (*p* = 0.038). For postoperative seizures preoperative tumor size (*p* < 0.001), sensomotory deficit (*p* = 0.004) and sphenoid wing location (*p* = 0.032) were independent risk factors. In terms of postoperative status epilepticus; sphenoid wing location (*p* = 0.022), tumor volume (*p* = 0.045) and preoperative seizures (*p* < 0.001) were independent risk factors. Postoperative seizures lead to a KPS deterioration and thus an impaired quality of life (*p* < 0.001). Late seizures occurred in 43% of patients with postoperative seizures. The small sub-cohort of patients (2.7%) with a STAMPE2 score of more than six points had a significantly increased risk for seizures (*p* < 0.001, total risk 70%). We concluded that besides distinct risk factors, high scores of the modified STAMPE2 score could estimate the risk of postoperative seizures. However, it seems not transferable to our cohort

## 1. Introduction

With a prevalence of almost one-third among brain tumors, meningiomas are among the most frequent of all intracranial neoplasms [1], most likely deriving from the meningothelial cells of the arachnoid layer according to the definition of the WHO [2]. The most common cranial localization is convexity followed by scull base localization with twofold higher incidence of higher grade meningiomas on the convexity [2,3]. About one-quarter of meningioma patients suffer from seizures according to population-based studies [4]. According to other studies, the seizure rate in meningioma patients (29–60%) is even higher than in primary glioblastoma (29–49%) or brain metastasis (20–35%), which is surprising since meningiomas are extra-axial tumors [5]. To date, the high seizure rate in meningioma patients has been a matter of debate with regard to the use of prophylactic antiepileptic drugs (AED).

In this context, very recent studies have aimed to identify risk factors for pre- and postoperative seizures. According to a recent meta-analysis, late seizures, which are defined as seizures occurring later than seven days after surgery, seem to present even more frequently than early ones [6]. Only a few studies involving a limited number of patients have addressed late seizures so far [7,8,9,10,11,12]. One of the largest retrospective studies investigating 779 patients who underwent surgery for cranial meningiomas consolidated the identified risk factors for postoperative seizures in the STAMPE2 score. However, this score has not been validated on an independent cohort yet. It is still not clear as to whether the administration of postoperative AED can reduce the occurrence of new postoperative seizures [13,14,15,16]. A promising study addressing this topic, the STOP’EM trial, is still recruiting [17].

Our retrospective study sought to identify risk factors for pre- and postoperative seizures with a special focus on late postoperative seizures and including and evaluating the STAMPE2 score in a modified version.

## 2. Results

### 2.1. Patient Cohort

Preoperative patient characteristics are summarized in Table 1; postoperative patient characteristics are summarized in Table 2. In our cohort of 420 patients, the female to male ratio was 2 (282 females and 138 males). The WHO grade at primary resection was grade I in 244 patients, grade II in 164 patients, and grade III in seven patients. The median age at the time of first operation was 56 years (mean: 55.9 years, range 16–86 years). Forty-four patients experienced recurrence after a median of 32 months (mean: 42.6 months, range 3-154 months). The median follow-up period was 21 months (mean: 28.9 months, range: 0–286 months). Median tumor volume was 23.5 cm^3^, interquartile ranges 8.65–43.3 cm^3^ (25–75th percentile) and 4.5–80.2 cm^3^.

**Figure 1:**
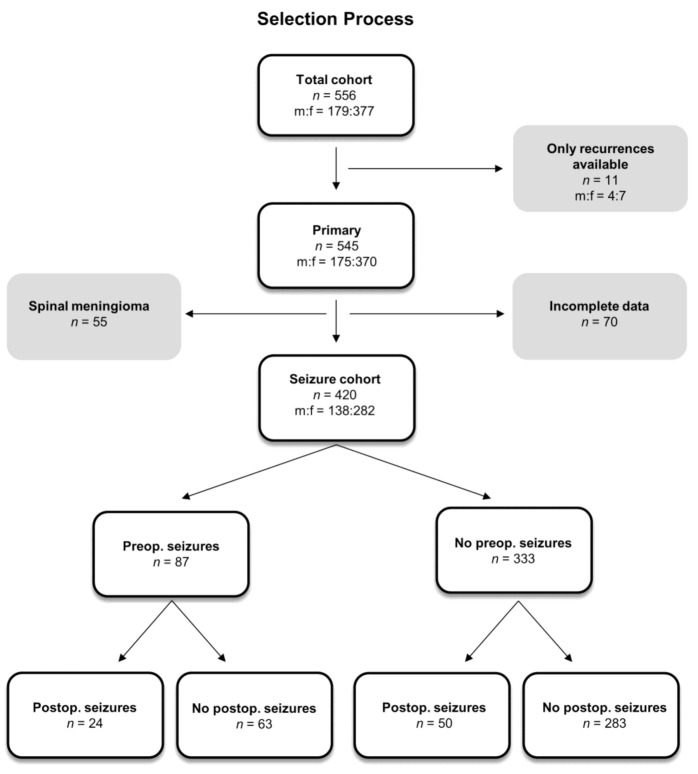
Flow diagram summarizing the selection process and giving an overview of the course of disease for patients with or without preoperative seizures.

Power analyses for pre- and postoperative seizures reached a power of 0.99, for postoperative status epilepticus it was 0.75. Of 420 patients in the primary situation with available data, preoperative seizures occurred in 87 (20.7%). Of those 333 patients who had no preoperative seizures, 283 patients (85%) remained seizure-free. Of the 333 patients with no preoperative seizures, 50 patients developed new postoperative seizures (15%; 11.9% of the whole population). In the group with preoperative seizures, 24 of 87 patients (27.6%; 5.7% of the whole population) had ongoing seizures, whereas 63 patients became seizure-free after operation (Figure 1). There was a significant association between pre- and postoperative seizures (Fisher’s test, *p* = 0.001). Localization of the cohort is summarized in Table S1.

### 2.2. Risk Factors for Preoperative Seizures

For preoperative seizures, the investigated risk factors are summarized in Table 3. Of all investigated factors, non-skull base location (*p* = 0.018), temporal location (*p* = 0.002), edema (*p* = 0.001) and male gender (*p* = 0.008) were significantly associated with a higher preoperative seizure rate in univariate analyses. Multivariate analyses of all factors that appeared with significance in univariate analyses (*p* < 0.25) were performed, respectively. After logistic regression, temporal location (*p* = 0.038) and preoperative edema (*p* = 0.039) remained as independent risk factors for preoperative seizures.

### 2.3. Risk Factors for Postoperative Seizures

For postoperative seizures, the risk factors are summarized in Table 4. During univariate analysis, the following factors showed an influence on postoperative seizures: Preoperative KPS (*p* = 0.020), tumor size (*p* = 0.036), sphenoid wing location (*p* = 0.045), sensomotory deficit (*p* = 0.022), speech disturbance (*p* = 0.032), age (*p* = 0.027), recurrence (*p* = 0.006), presence of preoperative seizures (*p* = 0.010), and preoperative AED use (*p* = 0.018). Multivariate analyses of all factors that appeared with significance in univariate analyses with *p* < 0.25 were performed. After logistic regression, only tumor size (*p* < 0.001), sensomotory deficit (*p* = 0.004) and sphenoid wing location (*p* = 0.032) remained as independent risk factors.

### 2.4. Risk Factors for Postoperative Status Epilepticus

For postoperative status epilepticus, considering the risk factors noted previously [18], they are further summarized in Table 5. During univariate analysis, sphenoid wing location (*p* = 0.043), re-craniotomy due to bleeding or swelling (*p* = 0.027), recurrence (*p* = 0.002), preoperative seizures (*p* < 0.001), and preoperative AED use (*p* < 0.001) were predictors for postoperative status epilepticus. After logistic regression including the risk factors that came out with *p* < 0.25 in univariate analyses, tumor volume (*p* = 0.045), preoperative seizures (*p* < 0.001) and sphenoid wing location (*p* = 0.022) remained as independent risk factors.

### 2.5. Early and Late Postoperative Seizures

**Figure 2:**
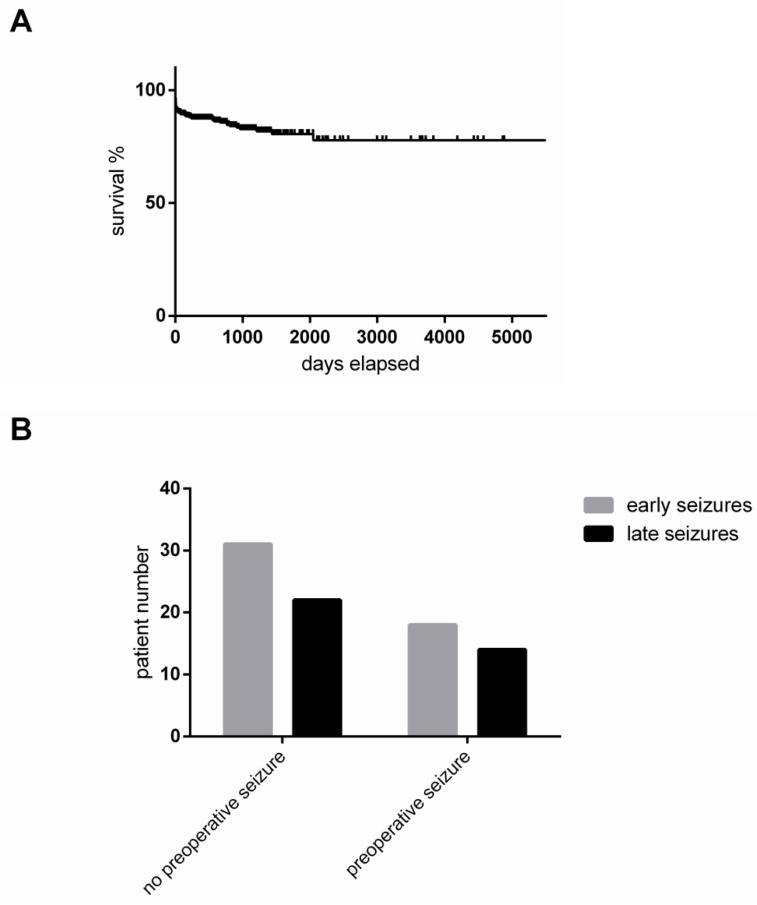
(**A**) Kaplan–Meier curve illustrates the seizure-free survival in our cohort. (**B**) Early versus late postoperative seizures in relation to the presence of preoperative seizures. Likelihood ratio: *p* = 0.8396; Pearson: *p* = 0.8395.

We defined early postoperative seizures as those that occur within the first seven days after operation. In total, 57% of all patients with postoperative seizures showed early postoperative seizures, while 43% experienced their first seizure more than seven days after the operation and thus were classified as demonstrating late seizures. Seizure-free survival of cranial meningiomas is depicted in Figure 2A. Seizure-free survival was not influenced by the onset of preoperative seizures in our cohort (Figure 2B).

### 2.6. Evaluation of the STAMPE2 Score

**Figure 3:**
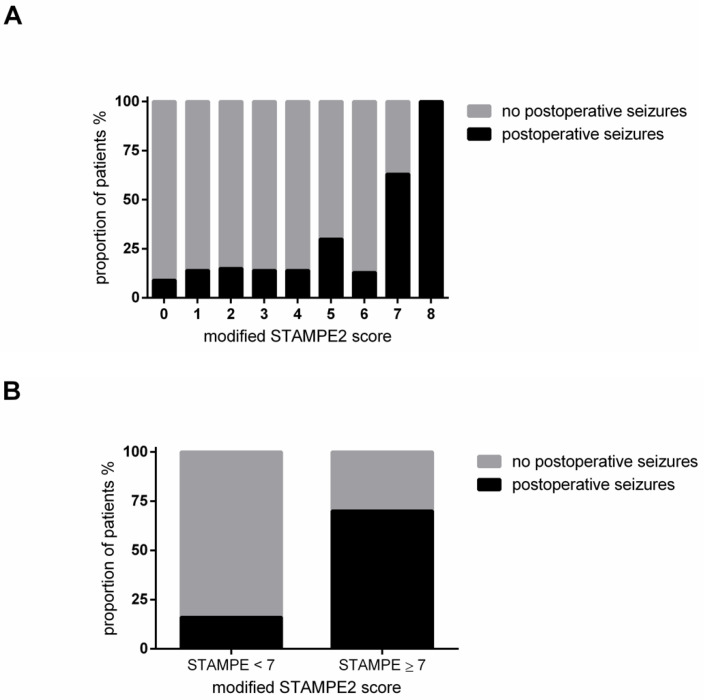
(**A**) Relative occurrence of postoperative seizures in the total cohort in relation to the modified STAMPE2 score. (**B**) Comparison of the occurrence of postoperative seizures for patients with a modified STAMPE2 score of seven points or more as compared with lower scores, Pearson: *p* < 0.001: The left column (modified STAMPE2 < 7) represents 97.3% of all patients, 57 patients with and 304 patients without seizures, the right column represents the remaining 2.7% of all patients, seven patients with seizures and three patients without seizures.

The distribution of our modified STAMPE2 is depicted in Figure 3A. Only few patients achieved high modified STAMPE2 scores in our cohort. When stratifying patients into two groups at six points or higher, a highly significant difference in the occurrence of postoperative seizures between the two groups was noted (Pearson Chi^2^: *p* < 0.001). Patients with a modified STAMPE2 score of less than seven points only had a 16% risk for postoperative seizures, whereas patients with a score of seven or more points had a risk of 70% for developing postoperative seizures (Figure 3B). Seizure-free survival and thus the occurrence of either early or late seizures did not correlate with the STAMPE2 score (Pearson Chi^2^: *p* = 0.0624).

### 2.7. Postoperative Seizures and Karnofsky Performance Score

Postoperative seizures led to a significant impairment of postoperative KPS (likelihood ratio: *p* = 0.001; Pearson: *p* < 0.001).

## 3. Discussion

### 3.1. Seizure Rate

Seizures are one of the three most common clinical symptoms observed in meningioma patients besides (and after) headache and neurological deficits [15]. Operation is typically the optimal treatment of choice for resolving those symptoms. In our retrospective cohort, 20.7% of the participants reported preoperative seizures, which is a similar high rate when compared with population-based data from France [4] and other recently published data showing an incidence of greater than 20% in the UK and the US [11,19]. However, other studies have reported preoperative seizure rates lower than ours [10,12,20]. The different incidence rates in the literature might be explained by regional differences in incidental asymptomatic but progressive meningiomas undergoing surgery due to a higher number of cranial MRIs performed for other reasons. According to our data, the biggest problem is not ongoing seizures that occur only in 5.7% of the whole population but new postoperative seizures occurring in 15% of all patients without preoperative seizures, which is lower than the 19.4% reported by Wirsching et al. [9], but remarkably higher than for example the 5.1% reported by Chozick et al. [21].

### 3.2. Risk Factors for Pre- and Postoperative Seizures

Defining risk factors for postoperative seizures was the aim of many recent studies as summed up by a meta-analysis conducted by our group [6]. In our cohort, edema and temporal location predicted preoperative seizures, a finding that has been echoed by several other groups recently [11,12,15,19,22,23,24,25,26,27]. Edema, which frequently occurs in meningiomas, still seems to somehow drive preoperative structural epilepsy. In our cohort, only tumor size, sensomotory deficit and sphenoid wing location were independently associated with the occurrence of postoperative seizures in multivariate analyses. Tumor size has been shown to be an risk factor already beginning with 8 cm^3^ in a different cohort [20]. Other groups could show that a diameter of >4 cm had significant influence on the occurrence of postoperative seizures [23,24]. Tumor location has been shown to have significant influence on the occurrence of postoperative seizures before [9,21,22,28,29,30]. Even though various locations have been published before, our data suggests that only sphenoid wing is relevant at least in our cohort. We were not able to show any advantage of the administration of AED in our retrospective setting. Despite the high rate of seizures that persist after operation in patients with preoperative seizures, such an observation has only rarely been addressed by other groups before [28,30]. Interestingly, swelling or bleeding/re-bleeding were not associated with postoperative seizures even though one would expect this. The effect might be masked by the high percentage of 43% of patients that experienced seizures after more than 7 days postoperatively.

### 3.3. Risk Factors for Status Epilepticus

Status epilepticus is associated with higher morbidity and mortality rates [31] and is a serious complication after meningioma surgery. According to our data, postoperative status epilepticus is independently associated with preoperative seizures, sphenoid wing location and tumor volume when setting the cut-off for inclusion in multivariate analyses at 0.25. Like for postoperative seizures, our data suggest that patients with tumors in this specific location as well as high tumor volumes and those with pre-operative seizures require special attention and maybe even need an increase in their medication dose after surgery. To our knowledge, we are the first group to report this special association with the rare complication of status epilepticus. Studies with larger cohorts need to re-evaluate these findings since the power for our cohort is only 0.75 for postoperative status epilepticus.

### 3.4. Time of Postoperative Seizure Onset

There are numerous retrospective studies mostly published during the past five years addressing risk factors for postoperative seizures. The onset of seizures after operation is very different; thus, we defined early seizures as those occurring in the first seven days after operation, while all others were defined as late seizures. Interestingly, a relatively high percentage of 43% of patients with postoperative seizures experienced late seizures, and this occurred in a regular postoperative management scenario out of the primary hospital. It is our opinion that these seizures are more dangerous and widely underestimated. To our knowledge, the first study reporting on seizure-free survival was the recent study by Islim et al. [11]. In our cohort, we could not define different risk factors for early and late postoperative seizures.

### 3.5. Evaluation of Modified STAMPE2 Score

Finally, it is very important to have a reliable scoring system to preoperatively assess the risk level for postoperative seizures so as to manage the patients better in the future. For that purpose, the STAMPE2 score was established by Wirsching et al. [9]. To our knowledge, we are the first group to evaluate the application of this valuable scoring system in an independent population. We had to modify the score, leaving out postoperative EEG since this is not performed in our clinic in a regular manner. Ultimately, we showed that patients with a score of seven points or more had a 70% risk for postoperative seizures, which is significantly higher than the risk for patients with a lower score. Only a very small subgroup of 2.7% of the patients reached a score of seven or higher in our total cohort. Still, the finding emphasizes the reliability of this well-elaborated score but only in a rare constellation in our cohort. However, not one single factor of all published by Wirsching et al. did show significant influence on postoperative seizures or status epilepticus in multivariate analyses in our cohort. That raises the question, whether single center studies can define a score to estimate patients at high risk for postoperative seizures.

### 3.6. Limitations

With a relatively large proportion of WHO grade II meningiomas our population differs from the reported distribution [2]. Thus, the transfer off our data to the general population might be limited. The retrospective character and the single center design of our study limits the informative value in some respects. Furthermore, we had to exclude the relatively high number of 70 patients due to incomplete data. The male to female ratio of 3:4 in the excluded patients differed from the main cohort. Multivariate data addressing the risk for postoperative status epilepticus is based on a relatively low power of 0.75 (Exact Agresti–Coull test).

## 4. Materials and Methods

### 4.1. Patient Data

We retrospectively analyzed the data of a cohort of 556 patients (377 females and 179 males) who underwent meningioma resection at our hospital. Eleven Patients of whom only recurrent tumors were treated in our hospital were excluded from further evaluation. Moreover, 55 spinal cases and 70 patients with incomplete data were excluded as well (Figure 1). Preoperative seizures, preoperative edema, tumor localization, tumor size, histological brain invasion, and the administration of pre- or postoperative AED (Benzodiazepines, Valproic Acid, Lamotrigine, Levetiracetam) was correlated with the occurrence of postoperative seizures and the occurrence of postoperative status epilepticus. The cutoff for early versus late seizures was seven days after surgery. For status epilepticus, we applied the International League Against Epilepsy (ILAE) definitions (AS and FR) as follows: 5 min for generalized tonic-clonic seizures, 10 min for focal seizures, 10 to 15 min for absence seizures. Furthermore, histological subtype, Ki67-proliferation rate, mitosis count per 10 high-power fields, World Health Organization (WHO) grade, according to the 2016 WHO classification and epidemiological data like age and gender were investigated as well. Tumor volumes were measured on T1 weighted MRI images and edema was re-evaluated as suggested by Wirsching et al. on T2 weighted MRI images [9]. All WHO grades II and III meningiomas were histologically re-evaluated by at least two neuropathologists as published before in the same cohort [32]. Histological re-evaluation was performed with special attention paid to mitosis and histological brain invasion. As surgical parameters, brain invasion defined by lacking an arachnoid layer and the Simpson score [33] were assessed. We also investigated the influence of repetitive operations in bifrontal tumors intentionally operated in two attempts, postoperative wound infections, re-operations due to bleeding or swelling, Karnofsky Performance Score (KPS) and postoperative irradiation.

### 4.2. Evaluation of STAMPE2 Score

Applying the STAMPE2 score [9], as published, was not achievable in our retrospective cohort and would not correspond to clinical practice since we conduct postoperative electroencephalography (EEG) only in selected patients in our hospital. Thus, we decided to leave out the factor “epileptiform potentials on postoperative EEG,” which was awarded two points in the initial study of Wirsching et al. A modified STAMPE2 score was applied to our cohort as follows: sensorimotor deficit (one point), tumor progression (one point), age of less than 55 years (one point), major surgical complication (two points), preoperative epilepsy (two points) and edema (one point). Our modified score thus had a range from zero to eight points.

### 4.3. Statistical Analysis, Software

Statistical analysis and figure editing were performed using JMP 14.0 software (SAS Institute, Cary, NC, USA), SPSS Version 25 (IBM Corp., Armonk, NY, USA), GraphPad Prism 6 (GraphPad Software Inc., La Jolla, CA, USA), and the open-source GIMP2 program. Evaluation of the immunohistochemical preparations was performed using a BX50 light microscope (Olympus, Tokyo, Japan). Measurement of tumor volume was performed using the SmartBrush tool of the Brainlab Elements software (Brainlab AG, Munich, Germany). A significance level of alpha = 0.05 was chosen for all tests (*p* = 0.05–0.01 → *; *p* < 0.01–0.001 → **; *p* < 0.001 → ***). For power analyses the Exact Agresti–Coull test was applied with a Null difference of 0.05. Survival analyses were performed using Kaplan–Meier analyses. In order to compare the survival curves, we used Wilcoxon and log-rank tests for censored data. For univariate correlations, Pearson’s test and likelihood ratio were used for dichotomized nominal ratios and median test was used for nonparametric testing of linear variables. For multivariate analyses, linear logistic regression was conducted. Parameters were checked for collinearity before inclusion, while the cut-off was set at 0.25, respectively.

## 5. Conclusions

Seizures and even status epilepticus after meningioma surgery are more likely to occur if the following risk factors are present: very large tumor size and/or sphenoid wing location. These patients need special attention in the postoperative management after meningioma surgery. The modified STAMPE2 score could predict postoperative seizures when reaching very high scores, however, the score seems not transferable to our cohort in terms of the included risk factors that did not reach significance in multivariate analyses.

## Figures and Tables

**Table 1:** Preoperative patient characteristics.

Factor	Preoperative Seizures		Total
	Yes	No	
Male	39 (28.3%)	99 (71.7%)	138
Female	48 (17%)	234 (83%)	282
Median Age	56 years	56 years	56 years
Skull-Base	21 (14.3%)	126 (85.7%)	147
Non-Skull-Base	66 (24.2%)	207 (75.8%)	273
Preoperative edema	58 (25.9%)	166 (74.1%)	224
Preoperative antiepileptic drug	79 (97.5%)	2 (2.5%)	81

**Table 2:** Postoperative patient characteristics.

Factor	Postoperative Seizures		Total
	Yes	No	
Male	28 (20.3%)	110 (79.7%)	138
Female	46 (16.3%)	236 (83.7%)	282
Median Age	61 years	54 years	56 years
Skull-Base	24 (27.3%)	123 (72.7%)	147
Non-Skull-Base	50 (18.3%)	223 (81.7%)	273
Preoperative edema	44 (19.6%)	180 (80.4%)	224
Preoperative seizures	23 (26.4%)	64 (73.6%)	87
Preoperative antiepileptic drug	22 (27.2%)	59 (72.8%)	81
**Modified STAMPE2 Score**			
0	3 (8.8%)	31 (91.2%)	34
1	9 (14.3%)	54 (85.7%)	63
2	11 (16.4%)	56 (83.6%)	67
3	10 (13.7%)	63 (86.3%)	73
4	9 (13.9%)	56 (86.1%)	65
5	13 (29.5%)	31 (70.5%)	44
6	2 (13.3%)	13 (86.7%)	15
7	5 (62.5%)	3 (37.5%)	8
8	2 (100%)	0 (0%)	2

**Table 3:** Risk factors for preoperative seizures.

Risk Factor	Univariate Analysis	Multivariate Analysis *p*-Value
	Pearson Chi^2^	
WHO grade > I	0.106	Not applicable
Brain invasion neuropathology	0.082	Not applicable
Brain invasion surgeon	0.433	
Preoperative KPS	0.793	
Tumor size	0.148	0.505
Non- skull base / skull base	0.018	0.425
Localization sphenoid wing	0.391	
Localization temporal	0.002	0.038
Edema	0.001	0.039
Sensomotory deficit	0.392	
Speech disturbance	0.182	0.638
Memory loss	0.323	
Age	0.922	
Male gender	0.008	0.212

**Table 4:** Risk factors for postoperative seizures.

Risk Factor	Univariate Analysis	Multivariate Analysis *p*-Value
	Pearson Chi^2^	
WHO grade > I	0.101	Not applicable
Brain invasion neuropathology	0.483	
Brain invasion surgeon	0.131	0.980
Preoperative KPS	0.020	0.761
Tumor size	0.036	<0.001
Non- skull base/skull base	0.745	
Localization sphenoid wing	0.045	0.032
Localization temporal	0.351	
Edema	0.132	0.668
Sensomotory deficit	0.022	0.004
Speech disturbance	0.032	0.152
Memory loss	0.642	
Age	0.027	0.963
Male gender	0.357	
Simpson grade III or higher	0.774	
Operation at two times	0.925	
Wound infection	0.604	
Re-craniotomy after bleeding/swelling	0.054	0.417
Intraoperative seizures	0.819	
Postoperative irradiation	0.316	
Recurrence	0.006	0.128
Preoperative seizures	0.010	0.576
Preoperative antiepileptic drug	0.018	Not applicable

**Table 5:** Risk factors for postoperative status epilepticus.

Risk Factor	Univariate Analysis	Multivariate Analysis *p*-Value
	Pearson Chi^2^	
WHO grade > I	0.123	Not applicable
Brain invasion neuropathology	0.276	
Brain invasion surgeon	0.658	
Preoperative KPS	0.162	0.119
Tumor size	0.129	0.045
Non- skull base/skull base	0.858	
Localization sphenoid wing	0.043	0.022
Localization temporal	0.303	
Edema	0.205	0.687
Sensomotory deficit	0.977	
Speech disturbance	0.154	0.079
Memory loss	0.385	
Age	0.400	
Male gender	0.326	
Simpson grade III or higher	0.873	
Operation at two times	0.157	Not applicable
Wound infection	0.440	
Re-craniotomy after bleeding/swelling	0.027	0.178
Postoperative irradiation	0.161	0.637
Recurrence	0.002	0.206
Preoperative seizures	<0.001	<0.001
Preoperative antiepileptic drug	<0.001	Not applicable

## Data Availability

The data presented in this study are available on request from the corresponding author. The data are not publicly available due to ethical restrictions.

## Supplementary Materials

The following are available online at https://www.mdpi.com/2072-6694/13/3/450/s1, Table S1: Influence of Location on pre- and postoperative seizure rate.

## References

[1] Ostrom Q.T., Gittleman H., Liao P., Rouse C., Chen Y., Dowling J., Wolinsky Y., Kruchko C., Barnholtz-Sloan J. (2014). CBTRUS Statistical Report: Primary Brain and Central Nervous System Tumors Diagnosed in the United States in 2007–2011. Neuro Oncol..

[2] Louis D.N., Perry A., Reifenberger G., von Deimling A., Figarella-Branger D., Cavenee W.K., Ohgaki H., Wiestler O.D., Kleihues P., Ellison D.W. (2016). The 2016 World Health Organization Classification of Tumors of the Central Nervous System: A summary. Acta Neuropathol..

[3] Kane A.J., Sughrue M.E., Rutkowski M.J., Shangari G., Fang S., McDermott M.W., Berger M.S., Parsa A.T. (2011). Anatomic location is a risk factor for atypical and malignant meningiomas. Cancer.

[4] Zouaoui S., Darlix A., Rigau V., Mathieu-Daudé H., Bauchet F., Bessaoud F., Fabbro-Peray P., Trétarre B., Figarella-Branger D., Taillandier L. (2015). Descriptive epidemiology of 13,038 newly diagnosed and histologically confirmed meningiomas in France: 2006–2010. Neurochirurgie.

[5] VanBreemen M.S.M., Wilms E.B., Vecht C.J. (2007). Epilepsy in patients with brain tumours: Epidemiology, mechanisms, and management. Lancet Neurol..

[6] Baumgarten P., Sarlak M., Baumgarten G., Marquardt G., Seifert V., Strzelczyk A., Rosenow F., Freiman T.M. (2018). Focused review on seizures caused by meningiomas. Epilepsy Behav..

[7] Connolly I.D., Cole T., Veeravagu A., Popat R., Ratliff J., Li G. (2015). Craniotomy for Resection of Meningioma: An Age-Stratified Analysis of the MarketScan Longitudinal Database. World Neurosurg..

[8] Waagemans M.L., Van Nieuwenhuizen D., Dijkstra M., Wumkes M., Dirven C.M.F., Leenstra S., Reijneveld J.C., Klein M., Stalpers L.J. (2011). A Long-term impact of cognitive deficits and epilepsy on quality of life in patients with low-grade meningiomas. Neurosurgery.

[9] Wirsching H.-G., Morel C., Gmür C., Neidert M.C., Baumann C.R., Valavanis A., Rushing E.J., Krayenbühl N., Weller M. (2015). Predicting outcome of epilepsy after meningioma resection. Neuro Oncol..

[10] Xue H., Sveinsson O., Bartek J., Förander P., Skyrman S., Kihlström L., Shafiei R., Mathiesen T., Tomson T. (2018). Long-term control and predictors of seizures in intracranial meningioma surgery: A population-based study. Acta Neurochir. (Wien).

[11] Islim A.I., Ali A., Bagchi A., Ahmad M.U., Mills S.J., Chavredakis E., Brodbelt A.R., Jenkinson M.D. (2018). Postoperative seizures in meningioma patients: Improving patient selection for antiepileptic drug therapy. J. Neurooncol..

[12] Wang Y.C., Chuang C.C., Tu P.H., Wei K.C., Wu C.T., Lee C.C., Liu Z.H., Chen P.Y. (2018). Seizures in surgically resected atypical and malignant meningiomas: Long-term outcome analysis. Epilepsy Res..

[13] Sughrue M.E., Rutkowski M.J., Chang E.F., Shangari G., Kane A.J., McDermott M.W., Berger M.S., Parsa A.T. (2011). Postoperative seizures following the resection of convexity meningiomas: Are prophylactic anticonvulsants indicated? Clinical article. J. Neurosurg..

[14] Zheng Z., Chen P., Fu W., Zhu J., Zhang H., Shi J., Zhang J. (2013). Early and late postoperative seizure outcome in 97 patients with supratentorial meningioma and preoperative seizures: A retrospective study. J. Neurooncol..

[15] Lieu A.S., Howng S.L. (2000). Intracranial meningiomas and epilepsy: Incidence, prognosis and influencing factors. Epilepsy Res..

[16] Yang M., Cheng Y.R., Zhou M.Y., Wang M.W., Ye L., Xu Z.C., Feng Z.H., Ma X.T. (2020). Prophylactic AEDs Treatment for Patients With Supratentorial Meningioma Does Not Reduce the Rate of Perioperative Seizures: A Retrospective Single-Center Cohort Study. Front. Oncol..

[17] (2017). Proceeding of the 2017 Meeting of the British Neurological Research Group, 2nd–3rd March. Br. J. Neurosurg..

[18] Trinka E., Cock H., Hesdorffer D., Rossetti A.O., Scheffer I.E., Shinnar S., Shorvon S., Lowenstein D.H. (2015). A definition and classification of status epilepticus—Report of the ILAE Task Force on Classification of Status Epilepticus. Epilepsia.

[19] Chen W.C., Magill S.T., Englot D.J., Baal J.D., Wagle S., Rick J.W., McDermott M.W. (2017). Factors Associated with Pre- and Postoperative Seizures in 1033 Patients Undergoing Supratentorial Meningioma Resection. Neurosurgery.

[20] Skardelly M., Rother C., Noell S., Behling F., Wuttke T.V., Schittenhelm J., Bisdas S., Meisner C., Rona S., Tabatabai G. (2017). Risk Factors of Preoperative and Early Postoperative Seizures in Patients with Meningioma: A Retrospective Single-Center Cohort Study. World Neurosurg..

[21] Chozick B.S., Reinert S.E., Greenblatt S.H. (1996). Incidence of seizures after surgery for supratentorial meningiomas: A modern analysis. J. Neurosurg..

[22] Chaichana K.L., Pendleton C., Zaidi H., Olivi A., Weingart J.D., Gallia G.L., Lim M., Brem H., Quiñones-Hinojosa A. (2013). Seizure control for patients undergoing meningioma surgery. World Neurosurg..

[23] Odebode T.O., Akang E.E., Shokunbi M.T., Malamo A.O., Ogunseyinde A.O. (2006). Factors influencing visual and clinical outcome in Nigerian patients with cranial meningioma. J. Clin. Neurosci..

[24] Kawaguchi T., Kameyama S., Tanaka R. (1995). Peritumoral edema and seizure in patients with cerebral convexity and parasagittal meningiomas. Neurol. Med. Chir..

[25] Hess K., Spille D.C., Adeli A., Sporns P.B., Brokinkel C., Grauer O., Mawrin C., Stummer W., Paulus W., Brokinkel B. (2018). Brain invasion and the risk of seizures in patients with meningioma. J. Neurosurg..

[26] Schneider M., Güresir Á., Borger V., Hamed M., Rácz A., Vatter H., Güresir E., Schuss P. (2019). Preoperative tumor-associated epilepsy in patients with supratentorial meningioma: Factors influencing seizure outcome after meningioma surgery. J. Neurosurg..

[27] Gupte T.P., Li C., Jin L., Yalcin K., Youngblood M.W., Miyagishima D.F., Mishra-gorur K., Zhao A.Y., Antonios J., Huttner A. (2020). Clinical and genomic factors associated with seizures in meningiomas. J. Neurosurg..

[28] Das R.R., Artsy E., Hurwitz S., Wen P.Y., Black P., Golby A., Dworetzky B., Lee J.W. (2012). Outcomes after discontinuation of antiepileptic drugs after surgery in patients with low grade brain tumors and meningiomas. J. Neurooncol..

[29] DeVries J., Wakhloo A.K. (1993). Cerebral oedema associated with WHO-I, WHO-II, and WHO-III-meningiomas: Correlation of clinical, computed tomographic, operative and histological findings. Acta Neurochir. (Wien).

[30] Zhang B., Zhao G., Yang H.-F., Wang D., Yu J.-L., Huang H.-Y. (2011). Assessment of risk factors for early seizures following surgery for meningiomas using logistic regression analysis. J. Int. Med. Res..

[31] Strzelczyk A., Ansorge S., Hapfelmeier J., Bonthapally V., Erder M.H., Rosenow F. (2017). Costs, length of stay, and mortality of super-refractory status epilepticus: A population-based study from Germany. Epilepsia.

[32] Baumgarten P., Gessler F., Schittenhelm J., Skardelly M., Tews D.S., Senft C., Dunst M., Imoehl L., Plate K.H., Wagner M. (2016). Brain invasion in otherwise benign meningiomas does not predict tumor recurrence. Acta Neuropathol..

[33] Simpson D. (1957). The recurrence of intracranial meningiomas after surgical treatment. J. Neurol. Neurosurg. Psychiatry.

