# Supplementary material for: Early and Late Postoperative Seizures in Meningioma Patients and Prediction by a Recent Scoring System
Source: Cancers (Basel). 2021 Jan 25;13(3):450. doi: 10.3390/cancers13030450 (PMC7865990; doi:10.3390/cancers13030450)
Supplement: Supplementary file 1 [file cancers-13-00450-s001.pdf]

Supplementary Materials

Early and Late Postoperative Seizures in Meningioma Patients and Prediction by a Recent Scoring System

Peter Baumgarten, Mana Sarlak, Daniel Monden, Andrea Spyrantis, Simon Bernatz, Florian Gessler, Daniel Dubinski, Elke Hattingen, Gerhard Marquardt, Adam Strzelczyk, Felix Rosenow, Patrick N. Harter, Volker Seifert and Thomas M. Freiman

Table S1. Influence of Location on pre- and postoperative seizure rate.

	Location	*n*	Preoperative Seizure Rate	Postoperative Seizure Rate
Non-Skull-Base	Frontal	115	22.6%	18.3%
	Fronto-parietal	11	36.4%	27.3%
	Fronto-temporal	12	25%	41.7%
	Parasagittal	3	0%	0%
	Temporal	12	41.7%	16.7%
	Temporo-parietal	2	100%	0%
	Parietal	25	24%	32%
	Parieto-occipital	10	20%	30%
	Tentorial	22	22.7%	0%
	Occipital	18	11.1%	22.2%
	Cerebellar	6	0%	0%
	Central	26	30.8%	7.7%
	Pre-central	4	25%	0%
	Post-central	3	66.7%	33.3%
	Pinealis	1	0%	0%
	Intraventricular	3	0%	33.3%
Skull-Base	Olfactory groove	18	16.7%	16.7%
	Fronto-basal	10	10%	10%
	Clinoidal	2	0%	0%
	Suprasellar	1	0%	0%
	Intrasellar	2	0%	0%
	Petrosal	8	0%	12.5%
	Foramen magnum	8	0%	0%
	Sphenoid wing	66	21.2%	27.3%
	Cerebellopontine angle	5	20%	0%
	Petroclival	12	0%	0%
	Spheno-orbital	11	0%	9.1%
	Sinus cavernosus	1	0%	0%
	Temporo-basal	3	66.7%	0%
